# Traumatic Rupture of Cystic Left Retroperitoneal Mass: An Atypical Presentation of Diffuse Large B-Cell Lymphoma

**DOI:** 10.7759/cureus.37797

**Published:** 2023-04-18

**Authors:** Joseph Genualdi, Tonny Orach, Shamon Gumbs, Gonzalo Ausqui, Brian Donaldson, Alexius Ramcharan

**Affiliations:** 1 Surgery, Columbia University Vagelos College of Physicians and Surgeons, New York, USA; 2 General Surgery, Harlem Hospital Center, New York, USA; 3 Surgery, Columbia University College of Physicians and Surgeons, Harlem Hospital Center, New York, USA; 4 Surgery, Harlem Hospital Center, New York, USA

**Keywords:** non-hodgkins lymphoma, trauma, splenic rupture, cystic retroperitoneal mass, diffuse large b cell lymphoma

## Abstract

Diffuse large B-cell lymphoma (DLBCL) is the most common type of non-Hodgkin lymphoma. Though the presentation is diverse, patients typically have a history of “B” symptoms and lymphadenopathy in areas such as the neck, mediastinum, or abdomen. However, a growing body of evidence suggests DLBCL can present as a cystic mass in diverse tissues. We present the case of a large cystic left retroperitoneal mass of unknown origin in a patient subsequently diagnosed with DLBCL. The diagnosis was obtained via percutaneous biopsy of the cystic mass in preparation for surgical excision. Upon diagnosis, surgical intervention was aborted, and the patient was started on chemotherapy treatment. However, four weeks into her treatment, she slipped and fell while in the bathroom and presented to the emergency department in shock with a computed tomography (CT) scan suggestive of splenic rupture. She underwent emergent splenectomy and resection of the cystic mass. She was discharged on postoperative day 7 and is currently continuing with outpatient chemotherapy. The presentation of DLBCL is notoriously diverse, however, this patient represents a unique presentation that adds to a growing body of literature suggesting DLBCL can present as a cystic mass. Pathological diagnosis should be obtained in all patients with cystic lesions of unknown origin before any surgical intervention to avoid unnecessary surgery and provide an optimal management plan.

## Introduction

Diffuse large B-cell lymphoma (DLBCL) is the most common type of non-Hodgkin lymphoma (NHL). The clinical presentation varies depending on the subtype and stage, however, patients historically present with the “B” symptoms. A relatively rare subtype of DLBCL that is associated with chronic inflammation was recognized by the World Health Organization lymphoma classification as a distinct entity. It presents as a mass in the setting of chronic inflammation and/or growth in the wall of a pre-existing cyst. Several cases of these entities have been reported in literature associated with pyothorax, splenic cysts, hydrocele, atrial myxoma, metallic-implant wear debris, thymic cyst, renal cyst, cranial arachnoid cyst, and ovarian cyst [1-7]. We present a case of de novo large left retroperitoneal cystic DLBCL in the absence of any chronic inflammation or pre-existing cyst. The patient was initially managed non-operatively, awaiting chemotherapy. However, patient subsequently suffered from blunt trauma requiring emergent splenectomy and excision of lesion. To our knowledge, this is the first case to be reported adding to the already diverse presentation of this disease.

## Case presentation

**Figure 1:**
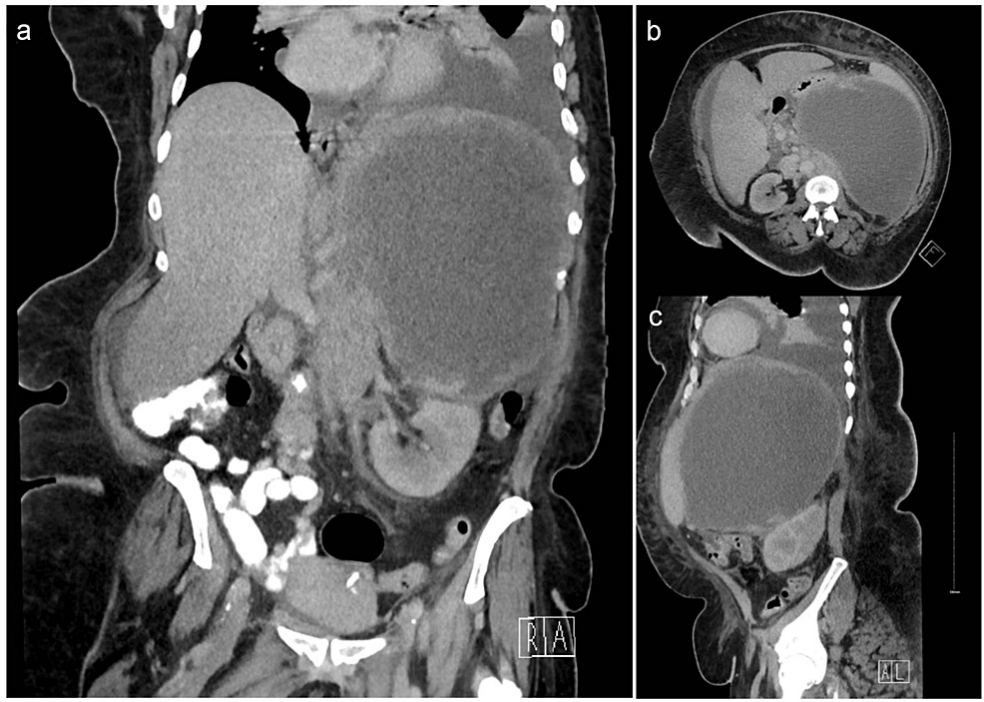
CT abdomen/pelvis with IV contrast with coronal (A), axial (B), and sagittal (C) views of the cystic retroperitoneal mass demonstrating displacement of retroperitoneal and intra-peritoneal structures. CT: computed tomography; IV: intravenous

A 53-year-old African American woman with a past medical history of chronic iron deficiency anemia, asthma, hypertension, and type 2 diabetes presented to our emergency department with a one-month history of fatigue, shortness of breath, and left-sided abdominal/flank pain. These were also associated with new-onset subjective fevers and diarrhea, which she attributed to her chronic anemia. On presentation, she was tachycardic (up to 111 bpm), with mild tenderness in the left upper abdominal quadrant and a notable large soft palpable mass. Labs were significant for leukocytosis (WBC 16 x 10^9^/L) and microcytic anemia (Hgb 8.3 x 10^12^/L). A computed tomography (CT) abdomen/pelvis was obtained and showed a large, predominantly cystic, left retroperitoneal mass measuring 21 x 15.5 x 22 cm with thickened, enhancing walls (1.6 cm thickness) associated with displacement of the left kidney and stomach. Multiple enlarged retroperitoneal/para-aortic lymph nodes were also noted (Figure 1).

A multidisciplinary meeting was held regarding the most appropriate management course. The decision was made to proceed with a percutaneous biopsy of the left retroperitoneal cystic mass. Interventional radiology (IR) performed core needle sampling of solid components for surgical pathology and drainage of the cystic component for cytology (1.4 L of salmon-colored fluid was drained). The patient’s symptoms improved markedly after the drainage.

The tumor markers CEA, CA 19-9, as well as amylase, and mucin were within reference ranges. Cystic fluid cultures were negative for any growth. The fluid sample for cytology was inconclusive due to the abundance of necrotic cell debris and the absence of viable cells. The final pathology of the cyst wall was significant for CD 45+ and CD 20+ necrotic cells consistent with large B cell lymphoma.

Surgical excision of the mass was deemed unnecessary since the patient’s symptoms had improved significantly after the drainage. The patient was referred to Medical Oncology to be initiated on chemotherapy.

**Figure 2:**
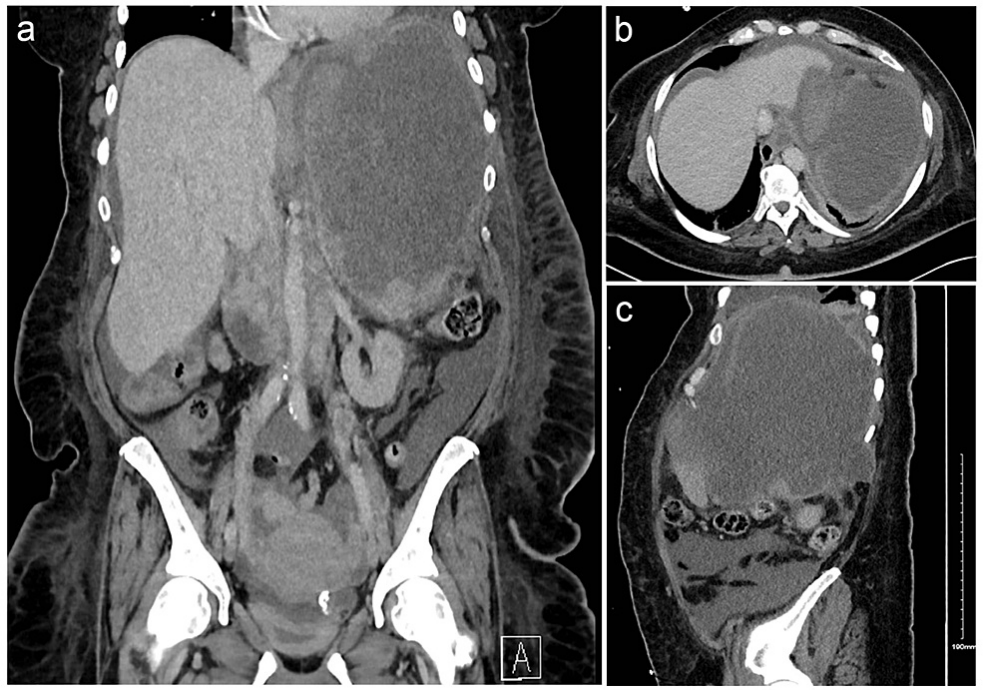
CT abdomen/pelvis with IV contrast, coronal (A), axial (B), and sagittal (C) views on re-admission demonstrating a large heterogeneous cystic septated retroperitoneal mass in the left upper quadrant causing significant mass effect on adjacent organs. The mass is poorly defined anteriorly with increased perisplenic ascites making rupture difficult to exclude. CT: computed tomography; IV: intravenous

After receiving the first cycle of her chemotherapy (about four weeks after discharge), the patient sustained a fall while in the bathroom hitting the left upper abdominal quadrant. She was brought to our hospital with generalized abdominal pain as a trauma activation. A CT abdomen/pelvis done after resuscitation was suggestive of a rupture of the cystic lesion/spleen due to increased ascites and/or hemoperitoneum (Figure 2).

She was taken emergently to the operating room for an exploratory laparotomy. Intraop, there was gross hemoperitoneum, extensive left upper quadrant desmoplastic reaction was noted, a ruptured spleen with areas of necrosis, and a large cystic cavity lined with tumor. The decision was made to perform a splenectomy with en bloc resection of the cystic cavity. The surgical planes were altered due to the extensive desmoplastic reaction. A Jackson-Pratt (JP) drain was left in the splenic fossa. Pathology was still consistent with large B-cell lymphoma. Postoperatively, she was transferred to the surgical ICU (SICU). She was able to be extubated on postoperative day (POD) 1 and remained hemodynamically stable. Abdominal exam remained unremarkable postop, and JP drain output remained serosanguinous.

She developed transient neutropenia with an absolute neutrophil count of 270 and a fever of 102 F, therefore was started on prophylactic antibiotics. The leukopenia was resolved by POD 5 and was stepdown to the surgical floor given her stability. Her drain output continued to decrease, however, remained >50 ml. Drain fluid was sent to evaluate for amylase, and was elevated to >11,000 U/L. During this period, she remained afebrile, stable, and tolerated a regular diet.

She was discharged on POD 7 with the drain in place and a plan for removal in the clinic once the output has decreased. She continued her follow-up with Medical Oncology to facilitate outpatient chemotherapy. The JP drain had to remain in place for more than three weeks due to output > 50 ml/24 hours with elevated amylase indicating grade B postoperative pancreatic fistula. The drain eventually was dislodged, and on follow-up visits had no further complications.

## Discussion

DLBCL is the most common type of NHL and predominantly affects elderly male patients with a median presentation in the seventh decade of life. Patients typically present with a rapidly growing mass involving one or more lymph nodes in the neck, abdomen, or mediastinum with classic systemic “B” symptoms (weight loss, fever, night sweats). However, there is notorious variability in stage and presenting symptom(s). Twenty percent of patients have localized disease and one-third have disseminated disease at presentation [2,8].

Standard treatment for DLBCL is chemotherapy, usually a combination of anti-CD-20 monoclonal antibody rituximab, and cyclophosphamide, doxorubicin, vincristine, and prednisone (CHOP) therapy with numerous clinical trials showing increased overall survival without an increase in adverse events to adding rituximab to standard CHOP therapy [9].

DLBCL associated with chronic inflammation is a distinct entity that usually presents as a mass and/or growth within the wall of pre-existing cysts. All the reported cases of DLBCL growing within pre-existing cystic lesions were diagnosed after surgical removal.

Our patient had no known pre-existing cystic lesion or chronic inflammatory condition so a pathological diagnosis prior to surgical excision was deemed necessary. Once the diagnosis of DLBCL was made, surgical management was discussed in the context of relieving the mass effect and preventing future complications. Though not typically a component of DLBCL management, surgical resection has been reported to prevent secondary complications arising from mainly primary intestinal DLBCL and to achieve symptomatic relief [10]. Ultimately, surgical intervention was deemed unnecessary in this case due to reduced mass effect after percutaneous drainage and confidence in follow-up for outpatient chemotherapy. However, the patient suffered a fall after just one cycle of chemotherapy and presented in shock necessitating splenectomy and en bloc removal of the lesion. The patient was rediscussed at a multidisciplinary meeting following the surgery and despite the rupture of the lesion after the fall, her management was considered optimal, and is continuing with chemotherapy.

## Conclusions

The clinical presentation of DLCBL is diverse and several cases of DLBCL have been found within walls of pre-existing cystic lesions after surgical resection. This case adds to this body of literature and supports the finding that, though rare, hematological cancers must be on a differential diagnosis for patients with cystic masses of unknown origin. And thorough evaluation with a biopsy is warranted for masses of unknown origin in stable patients before surgical intervention is warranted. Ultimately, these patients will require chemotherapy which remains the standard of care and surgery may still be required for symptomatic relief for some patients.
